# Exploring How Media Influence Preventive Behavior and Excessive Preventive Intention during the COVID-19 Pandemic in China

**DOI:** 10.3390/ijerph17217990

**Published:** 2020-10-30

**Authors:** Liqun Liu, Jingzhong Xie, Ke Li, Suhe Ji

**Affiliations:** 1 Center for Studies of Media Development, Key Research Institute of Humanities and Social Sciences at Universities, Ministry of Education, Wuhan University, Wuhan 430072, China; liqunliu@whu.edu.cn; 2 School of Journalism and Communication, Wuhan University, Wuhan 430072, China; xjzhong1989@whu.edu.cn; 3 School of Foreign Languages, Central China Normal University, Wuhan 430072, China; jisuhe@mails.ccnu.edu.cn

**Keywords:** COVID-19, mass media exposure, social networking services involvement, preventive behavior, excessive preventive intention, PLS-SEM, multi-group comparison

## Abstract

In the context of global fighting against the unexpected COVID-19 pandemic, how to promote the public implementation of preventive behavior is the top priority of pandemic prevention and control. This study aimed at probing how the media would affect the public’s preventive behavior and excessive preventive intention accordingly. Data were collected from 653 respondents in the Chinese mainland through online questionnaires and further analyzed by using partial least squares structural equation modeling (PLS-SEM). Taking risk perception, negative emotions, and subjective norms as mediators, this study explored the impact of mass media exposure and social networking services involvement on preventive behavior and excessive preventive intention. Based on differences in the severity of the pandemic, the samples were divided into the Wuhan group and other regions group for multi-group comparison. The results showed that mass media exposure had a significant positive impact on subjective norms; moreover, mass media exposure could significantly enhance preventive behavior through subjective norms, and social networking services involvement had a significant positive impact on negative emotions; meanwhile, social networking services involvement promoted excessive preventive intention through negative emotions.

## 1. Introduction

From December 2019 China’s first novel coronavirus disease (COVID-19) case identified in Wuhan to the very recent 30 August 2020, the World Health Organization (WHO) reported there were nearly 25 million cases and 800,000 deaths in the world, distributed in six continents and more than 200 countries and regions [1], which has become the most serious crisis that the world needs to face. As a new type of coronavirus, COVID-19 has many uncertainties in the route of transmission and treatment. Additionally, available specific drugs and effective vaccines have not yet been discovered by now. Therefore, behavior change (behavior to prevent transmission and infection) is the only feasible intervention measure to combat this public health emergency [2]. Although different countries have launched strategic battles against the virus in different ways, there is no doubt that publicizing and encouraging the public to undertake effective preventive actions is one of the important measures. It is self-evident that the media plays a critical role in improving public health by keeping people well informed of the health information and encouraging people to take preventive measures [3]. In the Chinese mainland, Hubei lifted outbound traffic restrictions on 25 March, which meant life gradually returned to normal, meanwhile the prevention and control of the pandemic had transformed from the emergency state to a normalized state. Naturally, protective measures including personal protection may last for a long time, so the establishment and maintenance of public protection behavior become particularly important.

In the field of public health, scholars have studied the influential mechanism of health risk information in media on individual cognition, attitude, and behavior. It is generally believed that health risk information can help people understand and perceive risks, take preventive measures, and provide policy support for reducing or mitigating risks [4]. The public’s risk perception, which examines the judgments people make when they are asked to characterize and evaluate hazardous activities and technologies, plays a key role in the response to health emergencies, affecting public policies, and individual behaviors [5]. In addition, the information provided by the media may also affect people’s emotions and subjective norms, and then affect the public’s protective behavior. Related empirical studies have manifested this process in different media platforms, including mass media dominated by newspapers [6], magazines [7], radio [8], television [9], and social media platforms such as social networking sites [10], blogs [11] and instant messaging [12]. During a public health crisis, people can search for and access a large amount of information through various channels including traditional media, interpersonal communications, and new media [13]. This information is vital for properly framing the risk perception and promoting responses [14]. However, false and misleading information in the media, especially social networks, may also promote a false perception of public health risks [15]. Therefore, it is essential to study the impact of different types of media (mass media and social media) on public preventive behavior in the context of a pandemic situation.

In the actual risk communication about the outbreak of COVID-19 in China, due to the severity and impacts of the infectious disease, the influence of media on preventive behavior possesses distinctive characteristics. On the one hand, the Chinese mainland had implemented rigorous public pandemic prevention measures after the outbreak of the disease. Especially on 23 January, Wuhan closed outbound traffic from the city; meanwhile, strict community closure measures in Wuhan city were implemented on 17 February. To minimize possible face-to-face social interaction, people relied on various media to seek for information about shopping, living security, and the update of the pandemic. Compared with any previous public health events, media plays a greater part during this crisis. However, media contact intensity of public access to pandemic information varies according to the severity of the pandemic and strictness of implementation of preventive measures in different cities (for example, Wuhan had implemented more stringent and longer-term preventive measures compared with other cities). On the other hand, although the government and health institutions had repeatedly publicized the relevant preventive measures through the media, some people still tended to take extra protective measures beyond the recommendations, that was, “excessive preventive intention” in terms of personal protection. Specifically, excessive preventive behaviors may include excessive sterilization with alcohol, the use of face masks beyond the necessary safety protection (for example, only N95 or multi-layer face masks are acceptable), and making a panic purchase of preventive drugs with unproven effect. These behaviors may lead to negative impacts on personal health, and worse still cause unnecessary waste of pandemic prevention materials, especially in the case of unexpected and inadequate preparation. However, there is a lack of research on public excessive prevention behavior in similar public health events. Thus, based on the comparison between Wuhan city and other Chinese mainland cities, this study expected to explicate the influence of mass media and social media on the public’s preventive behavior and excessive preventive intention with consideration to the varying severity of the pandemic.

## 2. Literature Review and Development of Hypotheses

### 2.1. Health Behavior in Pandemic

For different risks, the public could take preventive actions accordingly. During the pandemic, governments and health care institutions issued guidelines for prevention. However, there are differences in these guidelines in different countries and regions. In this study, the recommendation behaviors issued by Chinese official health care institutions were adopted as standards of preventive behavior. Additionally, some people in the Chinese mainland implemented personal protective measures beyond the recommended standards. However, because of the practical limitations (such as the shortage of N95 face masks at the early stage of the pandemic, giving priority to the needs of medical staff), these measures may not be implemented or may only be people’s intention. Previous studies have paid more attention to how to promote protective measures [16,17], while excessive preventive intention is rarely mentioned. This study hoped to explore whether preventive behavior and excessive preventive intention are affected by the same factors and structures.

Some behavior theories have also been applied in health communication to identify factors affecting decision-making for public health behavior. The representative ones are the health belief model (HBM), extended parallel process model (EPPM), the theory of planned behavior (TPB) and social cognitive theory, etc. [18]. These theories extend their application in empirical studies on public health emergencies, including SARS [19], Ebola [20], MERS [21], and influenza [22]. Although these theories try to describe the change of health behavior from different perspectives, they generally share the core concept that individual factors can influence and maintain the decisions on health behavior, provided that information is available [23]. Particularly, the individual’s social psychology has a direct or indirect impact on health behavior. These variables are classified into three dimensions in meta-theory of health communication: (a) cognitive factors, including perceived risk, subjective norms, attitudes, self-image, and self-efficacy; (b) emotional factors, covering fear, sadness, affection, pleasure, trust, and empathy; and (c) social context factors, involving mutual understanding, cohesion and reciprocity, and collective efficacy [24]. The effects of three factors on health behavior are complementary and accumulative. Studies have shown that in a given environment, the more these factors play a role, the more likely they are to promote health behavior [25]. Although studies seek to elaborate on public health behavior from these three dimensions, it has not yet reached a consensus about effects of the theories in different situations. This study focused on some key individual’s social psychosocial variables (Table 1) that influence public health behaviors, namely risk perception, negative emotions and subjective norms, and explored their mediating roles in the influence of different media (mass media exposure and social networking services involvement) on health behaviors.

### 2.2. Media Activities

After reviewing a large number of studies on health communication, many scholars have reached a consistent view that exposure to media activities can affect public health behavior [30,31]. However, it only poses a limited effect on behavior [32]. In the past few decades, many hypotheses, theories, and models have emerged in the field of health communication to explain the relationship between media and public health behavior. Among them, the hypothesis of the influence of presumed influence hypothesis (IPI) examines the indirect effects of media influence and holds that perception of social norms is formed through the exposure to media information, and then the perception motivates people to observe the opinions accepted by the majority [33].

Previous studies suggest that interpersonal discussion, besides mass media, is also a vital channel to obtain and exchange health-related information [34]. That is the influential intensity of the mass media. The timing and context matched to the information gained from mass media can prompt people to realize the personal relevance of the information, and then trigger further discussion in social networks [35], which influences the follow-up behavior of discussants in interpersonal discussion [36]. Although the quality of communication via mass media might make a difference more than its quantity, the influential intensity of the mass media is more important and necessary in such a health emergency. Thus, mass media exposure will interact with social networking services involvement, and then put an effect on individual health behavior [26]. Hence, based on previous studies, we proposed the following hypothesis:

**Hypothesis** **1** **(H1a).**  *Mass media exposure has a positive impact on social networking services involvement.*

### 2.3. Risk Perception

Risk perception involves people’s subjective assessment of the probability that possible negative consequences or diseases might come up [5]. Tyler classified risk perceptions into the personal level and social level [37]. The former refers to the assessment of the serious effects of potential risks on the individual himself; while the latter associates with the elevation of risks to others or the whole society. Recent studies have shown that the media will considerably influence people’s sense of risk issues during the outbreak of public health emergencies [21,38]. There are two hypotheses to explain the impact of media on risk perception. The impersonal impact hypothesis argues that the risk information of mass media will initiate and strongly affect the social level of risk perception, while posing a weaker impact on the individual level of risk perception [38]. However, the distinct impact hypothesis emphasizes the impact of different types of media on PB. The Social Amplification of Risk Framework (SARF) believes that the media can play the role of a social amplifier, amplifying or weakening the public risk perception through agenda setting [39]. And mass media can advocate the risk more prominent, and the more people are exposed to information that causes increasing negative emotions, the more likely that they discuss it [40]. While the entertainment media (social media) causes people to judge a higher possibility of personal risk [41]. As a result, attention will move away from mass media to interpersonal communication for further judgments [42].

As a digital form of interpersonal communication, social media promotes the sharing of risk information and the flow of emotion. Some studies have demonstrated that social media interaction poses a greater influence on personal risk perception than exposure to risk information [42]. The underlying reason is that, compared with mass media, interpersonal communication is more interactive and individual and demands more resources during the mental processing of risk information [43]. During the pandemic, public information carried in mass media facilitates the spread of pandemic information and reports of preventive measures, which may weaken the risk perception of the public. However, social networking services involvement may enhance risk perception through discussions on pandemic situations. Therefore, we put forward the following hypotheses:

**Hypothesis** **1** **(H1b).**  *Mass media exposure has a negative impact on risk perception.*

**Hypothesis** **2** **(H2a).**  *Social networking services involvement has a positive impact on risk perception.*

According to the two-step process model of behavior change, the initial step of attitude change is that media exposure influences people’s cognitive beliefs (such as risk perception), which in turn causes certain behavior changes, and resultant behavioral change is the second step [44]. As a key predictor of health behaviors, RP is regarded as the core concept of health behavior theories, like HBM [45], protection motivation theory [46], and prevention adaptation process model [47]. Studies have posited that when people perceive the risk, they will actively take preventive health behavior [38,48]. Therefore, the following hypotheses were put forward:

**Hypothesis** **3** **(H3a).**  *Risk perception has a positive impact on preventive behavior.*

**Hypothesis** **3** **(H3b).**  *Risk perception has a positive impact on excessive preventive intention.*

### 2.4. Negative Emotions

In the risk communication practice, the role of emotion is often ignored until scholars confirm the potential psychological structure of risk in the public mind [5], which includes not only a rational judgment of risk but also strong emotions such as fear and anger [49]. The “risk-as-feeling” model and affect heuristics show that the cognitive system (risk assessment) interacts with the emotional system, and then affects behavior. Previous studies have proved that media framing highlights risks and consequently leads to public panic [50]; nevertheless, studies also have demonstrated that the mass media (especially the official mainstream media) pay increasing attention to risk communication, and curb a health crisis to reduce public panic by emphasizing solutions and successful containment measures [51]. Undoubtedly, it has become a form of appeasement and a tool to eliminate panic [52].

Negative emotions are more common in social media than news sites and blogs [53], because the public health crisis information on social media is usually constructed in an emotional way [54], which is more likely to evoke personal emotions through a vivid dramatic description of the risk. A recent study shows that the mainstream media in China mainly emphasize information about instrumental support and praise people or organizations, while the information which shows empathy to affected people, blames other individuals or government, and expresses worry about the pandemic is more active in the discussion taking place in social media during the early pandemic stage in the Chinese mainland [55]. Although discrete emotions have different roles in the formation of perceptual and behavioral outcomes [56], studies have shown that both fear and anxiety can encourage individuals to avoid or prevent a particular threat, thus prompting them to seek information that may be relevant to protective measures against the threat [57]. Therefore, in this study fear and anxiety were chosen as representatives of negative emotions to investigate their role in the relationship between media and health behavior. And we put forward the following hypotheses:

**Hypothesis** **1** **(H1c).**  *Mass media exposure has a negative impact on negative emotions.*

**Hypothesis** **2** **(H2b).**  *Social networking services involvement has a positive impact on negative emotions.*

Much persuasive evidence indicates that self-related emotions, like fear, anxiety, and anger, contribute greatly to people’s risk assessment and subsequent behaviors to control the risk [40]. The affect-as-information model assumes that complex evaluation will be made in a heuristic way based on individuals’ current emotional state, as long as this experienced emotional state is related to the evaluation target [58]. The Appraisal Tendency Framework (ATF) also shows that each emotion corresponds to a specific evaluation dimension, resulting in distinctive risk perception [56]. All the relevant theories and models above show that emotion can affect risk perception, and based on the above literature review, the hypothesis was formed:

**Hypothesis** **4** **(H4a).**  *Negative emotions have a positive impact on risk perception.*

In addition to influencing behavior results through risk perception, emotion can also directly cause preventive behavior [59]. However, some researchers have proposed that different emotions have different motivations and behavioral functions [60]. For example, fear can stimulate behavior which is aimed to solve or avoid problems [61]; meanwhile fear may also prevent people from participating in such behaviors when fear is strong [62]. Therefore, the relationship between fear and behavior is inverted U-shaped [63]. The significant effect of fear on preventive behavior has been demonstrated in many studies [64,65]. The very recent research has proved that when there is no alternative (such as an unexpected outbreak of an infectious disease), a high level of fear will encourage preventive behavior [21]. Additionally, in the extended study of EPPM theory, two kinds of emotions related to threat are conceptualized as fear and anxiety [66]. Compared with fear, anxiety originates from the uncertainty of threat and stimulates greater motivation for information seeking. Therefore, we believed negative emotions would affect the public’s preventive intention and behavior, and then proposed the following hypotheses:

**Hypothesis** **4** **(H4b).**  *Negative emotions have a positive impact on preventive behavior.*

**Hypothesis** **4** **(H4c).**  *Negative emotions have a positive impact on excessive preventive intention.*

### 2.5. Subjective Norms

Related empirical studies have conceptualized and operationalized normative perception to explore the role of normative effects on health behavior change, such as subjective norms [67] and social norms [68], etc. Among them, subjective norms were the most widely used and can be divided into descriptive norms and injunctive norms. The former refers to reflecting whether others have performed their actions; whereas the latter can be defined as individuals’ perception of what important others would approve or think one should do [69]. This study focuses on the impact of descriptive norms on preventive behavior, not only because descriptive norms have a more direct and significant impact on health behaviors than injunctive norms [70], but also because of the actual situation of the outbreak of pandemic is taken into account. During the pandemic, due to the extensive publicity of public health institutions in China, the public had already had very high injunctive norms on PB. They mainly wanted to obtain the degree of popularity of preventive behavior from the media, that is, descriptive norms.

In everyday life, the daily routine behaviors like observing others, talking with friends and family, learning public policies, and mass media consumption appear to help people to obtain and form normative perceptions [71]. Some studies have shown that exposure to health information in social media acts as a strong indicator to predict descriptive norms and injunctive norms [72], nevertheless, others have demonstrated that exposure to health information in WeChat has a negative predictive effect on users’ descriptive norms, meanwhile exposure to health information in WeChat has no significant impact on injunctive norm [12]. Thus, we addressed the following hypotheses accordingly:

**Hypothesis** **1** **(H1d).**  *Mass media exposure has a positive impact on subjective norms.*

**Hypothesis** **2** **(H2c).**  *Social networking services involvement has a positive impact on subjective norms.*

Previous studies have shown that subjective norms are considered as one of the key predictors of behavior in the theory of reasoned action (TRA) [67], the theory of planned behavior (TPB) [73], and the information-motivation-behavioral skills model (IMB) [74]. Moreover, it has been verified in various empirical studies on health behavior [12,72]. In particular, it demonstrates the strong influence of descriptive norms on skin cancer prevention [75], a healthy diet [76]. Based on the above research, we put forward the following hypothesis:

**Hypothesis** **5** **(H5).**  *Subjective norms have a positive impact on preventive behavior.*

### 2.6. Severity of Pandemic Situation

Geographical or physical resemblances to events are regarded as an important factor affecting event perception [77]. Some studies found that further distance between individuals and risk sources causes individuals to rate the risk at a higher level [78]. However, it is proved that the closer the public perception of the outbreak, the higher is the degree of concern and fear of the pandemic [79]. Although the diseases are very different and might have different impacts also on people’s imaginary and the conclusions of these studies are also still controversial, they show that the severity of the pandemic and the spatial distance of the outbreak have an impact on public psychology. In the early stage, Wuhan reported the first pandemic case, and Wuhan was identified as the most severely affected city in the Chinese mainland. After taking public preventive measures, such as temporary closure of outbound traffic from the city, temporary closure of communities, the public obtained information mainly through the media, especially through social media. Compared with other regions (OR), the public in Wuhan (WH) may be more dependent on social media and more vulnerable to the influence of social media. Therefore, we proposed the following assumptions:

**Hypothesis** **6** **(H6a).**  *Compared with other regions in the Chinese mainland, social networking services involvement has a greater impact on risk perception in Wuhan.*

**Hypothesis** **6** **(H6b).**  *Compared with other regions in the Chinese mainland, social networking services involvement has a greater impact on negative emotions in Wuhan.*

**Hypothesis** **6** **(H6c).**  *Compared with other regions in the Chinese mainland, social networking services involvement has a greater impact on subjective norms in Wuhan.*

**Figure 1:**
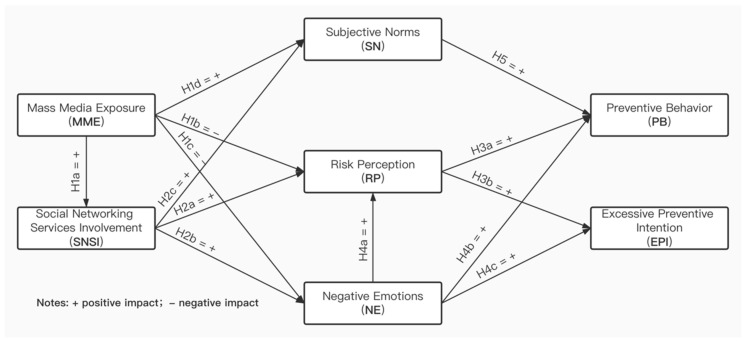
Research model.

Figure 1 presents the research model and hypotheses.

## 3. Method

### 3.1. Data Collection

#### 3.1.1. Participants

The participants were all from the Chinese mainland who lived in the Chinese mainland from the outbreak of the pandemic to the survey. Due to the requirements of pandemic prevention and control during that period, it was impossible to collect data from offline participants. Therefore, participants were recruited through online questionnaire survey platforms. Considering the number of participants and the coverage of cities, two online questionnaire platforms, Tencent [80] and Wenjuanxing [81], were selected. These two platforms have millions of samples, covering most of the Chinese mainland cities. At least 2500 people participated in the survey on the two platforms, and 711 complete questionnaires were collected.

#### 3.1.2. Material

The data were collected through an online questionnaire. The questionnaire was divided into two parts. The first part was the measurement of the variables involved in the research hypotheses. The second part was the personal information of the respondents. In addition to gender, age, education level, it also included the information about the city where the respondents lived during the outbreak of the unexpected disease, whether they were infected or were close contacts, whether they were front-line medical workers, etc.

In the present study, the constructs were measured through adapted items that were derived from previous research and modified to meet the requirements of this study. All the items were presented in the in the Appendix in detail. Items were scored on a 1–7 Likert scale, with 1 referring to least frequent or strongly disagree and 7 most frequent or strongly agree. The respondents filled out questionnaires based on their own ideas and experience in the past month. Measurements for most of the constructs in the study followed previous studies. Mass media exposure and social networking services involvement were measured using items derived from Li [26]. Four items of risk perception were adopted from Cho and Lee [82]. The measurement of negative emotions was modified following the research of So, Kuang, and Cho [57], Lagoe and Atkin [83], and Zhang et al. [84]. Two typical negative emotions, fear, and anxiety, were measured through six items. Subjective norms were measured with the scale of descriptive norms used by Park and Smith [85]. Measuring preventive behavior against coronavirus disease was taken from six typical preventive measures officially recommended by the Chinese center for disease control and prevention. This is also the most authoritative guide for protective behaviors in the Chinese mainland. After completing the evaluation of their own preventive behaviors, the respondents were further asked whether they believed that the preventive measures were sufficient to provide enough protection so as to assess the respondents’ excessive preventive intention.

#### 3.1.3. Procedure

Data collection was conducted in the Chinese mainland from 30 March to 5 April 2020. The reason why this period was selected was that Hubei lifted outbound traffic restrictions on 25 March, which meant all cities in the Chinese mainland including Wuhan city had gradually entered the stage of ongoing prevention and control. The medical face masks and other essential pandemic prevention supplies had been fully restored to normal and sufficient market supply to pave the way for the ongoing pandemic prevention. More importantly, there were no objective obstacles in the implementation of preventive behavior. Given the possibility of a domestic resurgence at any time, the period of data collection should be shortened as much as possible. Therefore, a five-day data collection was conducted from 30 March to ensure that all respondents in all regions were not affected by the pandemic outburst, and the data would reflect the attitude of respondents under the normal situation of pandemic prevention and control.

### 3.2. Data Analysis

The quality of the questionnaire was strictly controlled. The incomplete questionnaire was excluded from the valid questionnaire, and 711 questionnaires were finally collected. As the infected, close contacts, front-line medical workers may be significantly different from ordinary respondents in preventive behavior, negative emotions, etc., these samples were removed. Moreover, some inadequate questionnaires (such as the length of complete-time, sameness of answers) were eliminated, and finally 653 valid questionnaires were obtained. The study relied on data from 208 completed questionnaires from Wuhan, and 445 from other regions in the Chinese mainland. 51% of Wuhan respondents were female (N = 106), while 49% were male (N = 102). Wuhan respondents with age ranging from 18 to 30, and 30 to 40, both accounted for 36.1% (N = 75). 66.1% of respondents from other regions in the Chinese mainland were female (N = 294), while 33.9% were male (N = 151). Respondents from other regions in the Chinese mainland with age ranging from 18 to 30 accounted for 76.6% (N = 341). The samples in both groups exceeded the minimum required PLS-SEM sample size [86].

SmartPLS 3.3.2 (SmartPLS GmbH, Boenningstedt, Germany) [87], was employed to analyze the data, including the evaluating of the measurement model and structural model, conducting multi-group analysis (MGA), and importance-performance map analysis (IPMA). MGA is employed to examine whether the PLS model is significantly different between groups. In this study, MGA was used to test if the PLS model differs between the Wuhan group and other regions group. Importance-performance map analysis is an extended analysis approach in PLS-SEM, which embodies more abundant results. In a graphical representation, the IPMA contrasts the importance and the performance in the structural model. The importance, which is presented on the x-axis, is the representation of the unstandardized total effects; whereas, the performance presented in the y-axis refers to the average values of the latent variable scores, which is measured on a scale from 0 to 100 [86]. By adding a dimension to the analysis of PLS-SEM results, IPMA extends the standard results.

Compared with covariance-based structural equation modeling (CBM-SEM), PLS-SEM is more suitable for processing complex models [88], and a previous study has suggested that in terms of the assessment of reflective and formative constructs, PLS-SEM is a better choice [89]. Besides, PLS-SEM is a non-parametric SEM technique that is appropriate to conduct MGA [90].

## 4. Results

### 4.1. Common Method Bias

Common method bias (CMB) is caused by the measurement method rather than the causes or effects of the model; it is a common phenomenon in the context of PLS-SEM. However, the ignorance of the common method may artificially increase the level of convergent validity of the model being studied, which would lead to statistical error [91]. In this study, the full collinearity test proposed by Kock and Lynn was used to test whether the model has a common method bias [92]. The threshold value of the complete collinearity test is 3.3. When the coefficient value is lower than 3.3, the measurement model is not affected by CMB. The test results of each construct in this study ranged from 1.29 to 1.79, which indicates an absent concern for the CMB.

### 4.2. Descriptive Analysis

The descriptive statistical results of items and constructs were shown in Table 2. It can be seen from the results that the mean values of all the constructs of the Wuhan group were greater than those of other regions group; especially in excessive preventive intention, negative emotions, risk perception and subjective norms. In addition, the values of preventive behavior and subjective norms of the two groups were relatively high, indicating that the respondents’ self-evaluated preventive behavior was well implemented, and at the same time their belief in subjective norms was rather strong.

### 4.3. Assessment of Measurement Model

The measurement models indicate the relationships between constructs and indicator variables [86]. The assessment of the measurement models mainly includes the test of reliability and validity. There are some differences in the evaluation indexes between reflective measurement models and formative measurement models.

In this study, four aspects were considered to evaluate the reflective measurement models: indicator reliability, internal consistency, convergent validity, and discriminant validity. Generally, the indicator reliability is verified by the size of outer loading higher than the threshold value of 0.45 [93]. Internal consistency is measured by Cronbach’s α and composite reliability (CR). Cronbach’s α should surpass the recommendation of 0.7, and CR should be greater than 0.7 [94]. The average variance extracted (AVE) is used to guarantee the convergent validity, and the AVE should exceed the level of 0.5 [95]. Discriminative validity is confirmed by the heterotrait-monotrait (HTMT); moreover, a bootstrapping procedure with 5000 resamples obtains the HTMT value. And HTMT confidence interval does not include 1, which suggests that discriminative validity is acceptable [86], and the result was shown in Table 3. In the present study, the indicator reliability test revealed one invalid item- the item RP1 whose loading is 0.591. As a result, after removing item RP1, all the data achieved the required cutoff values, which indicated that criteria had been fulfilled. And the specific data were illustrated in Table 4.

To evaluate the formative model, we needed to investigate three aspects: convergence validity, collinearity issues, significance, and relevance of the formative indicators. Convergent validity is measured by redundancy analysis. Redundancy analysis is examined by the correlation between the formative construct and an alternative measure of the construct which uses a global single item [86]. The global single items of mass media exposure, social networking services involvement, and preventive behavior have been included in the questionnaire. The value of the correlation between the constructs shows convergent validity. Ideally, the value should be 0.80, or at least higher than the threshold value of 0.70 [86]. Collinearity issues are evaluated by the variance inflation factor (VIF). When VIF is below the threshold value of 5, it indicates the absence of the problematic collinearity issues. The significance and relevance of the formative indicators is the contribution of formative indicators to the construct. Whether or not the formative indicators are being removed depends on the outer weights and outer loadings as well as their theoretical significance. If the outer weight of the indicator is significant, it should be retained; if not, its outer loading should be tested. When the value of outer loading is greater than 0.5, it should be retained. If the outer weight is less than 0.5, the significance of outer loading should be further confirmed. If it is significant, it should be considered whether to retain it according to the theoretical value of the item. When the outer weight is non-significant, it should be deleted [86]. After the preliminary evaluation of the formative model, MME2 and MME5 did not meet the requirements. Theoretically, it may be due to the less frequent usage and small range of newspapers and radio compared to other media. According to the suggestion of Hair et al. and considering that the items in the subsequent multi-group comparison should be consistent, the two items were removed [86]. After removing the two items, all the results fulfilled the criteria. The specific data were shown in Table 5.

### 4.4. Measurement Model Invariance

Measurement invariance is a guarantee of the validity of multi-group analysis; it ensures that differences between groups are not caused by the content and/or meanings of the latent variables of different groups [86]. In SmartPLS 3.3.2 measurement invariance of composite models (MICOM) function can be employed to verify measurement invariance. MICOM includes three steps: step 1 involves the establishment and assessment of configural invariance; while step 2 focuses on the equality of a composite among different groups; furthermore, step 3 is an analysis of equality of composite mean value and variances [86]. However, these three steps are not independent but hierarchically intertwined instead. Step 1 and step 2 are the preconditions for measurement equivalence. If results from the previous two steps support measurement invariance, partial measurement invariance is verified, which allows further comparison of path coefficient estimates among different groups. Only when partial measurement invariance is verified and the results of equal mean values and variances among groups are confirmed can the pooled data analysis be run. The results of this study revealed partial measurement invariance, and the specific data were illustrated in Table 6.

### 4.5. Assessment of the Structural Model

**Figure 2:**
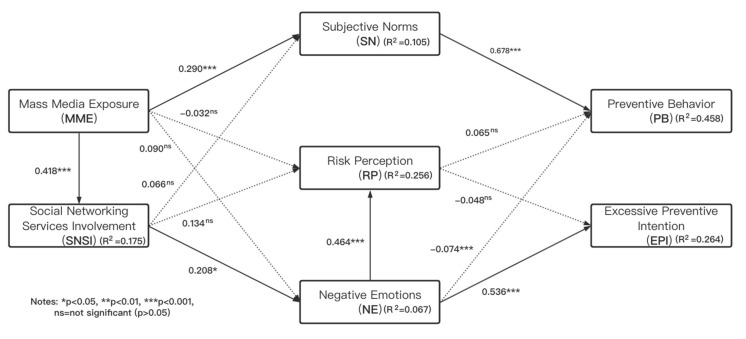
Results of the structural model analysis for the Wuhan group.

**Figure 3:**
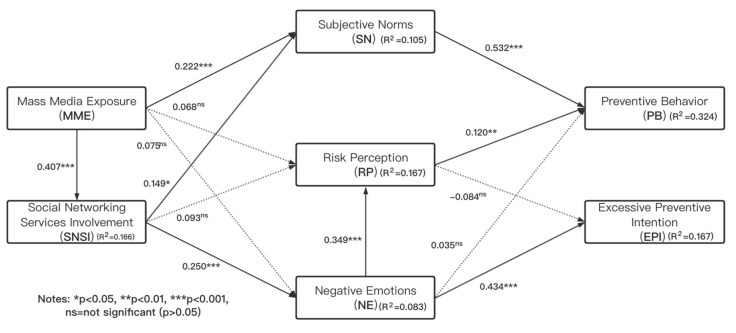
Results of the structural model analysis for the other regions group.

Because the results of MICOM confirmed the establishment of partial measurement invariance, it cannot proceed to pool the data. Therefore, the assessment of the structural models is performed separately. Generally, the assessment of PLS-SEM’s structural model usually needs to examine structural models for collinearity issues, the significance and relevance of the structural model relationships, the level of R^2^, the f^2^ effect size, and standardized root mean square residual (SRMR). VIFs are commonly used to assess collinearity. Each prediction structure is checked separately for each part of the structural model. The results showed that the maximum VIF value in the model was 1.343, which is less than the standard value of 5. To test the hypothesis, bootstrapping (5000 subsamples) was adopted to assess the significance of path coefficients in the structural models. The path coefficient and significance were shown in Table 7, Figure 2, and Figure 3. Since the coefficient of determination (R^2^ Value) represents the model’s predictive power, it is most commonly used to evaluate structural models in PLS-SEM. According to Chin [88], R^2^ values of 0.67, 0.33, and 0.19 are regarded as substantial, moderate, and weak, respectively. The results showed that for the Wuhan group, our model had moderate explanatory for preventive behavior ($R_{WH}^{2}$ = 0.458), and weak explanatory for excessive preventive intention ($R_{WH}^{2}$ = 0.264). For other region groups, our model had a moderate explanatory for PB ($R_{OR}^{2}$ = 0.324) and rather weak explanatory for excessive preventive intention ($R_{OR}^{2}$ = 0.167). The f^2^ is used to evaluate the predictive effects between particular constructs. According to Cohen [96], the guiding principle for the assessment of f^2^ is that 0.02, 0.15, and 0.35 represent the small, medium, and large effects of exogenous latent variables, respectively. In this study, the f^2^ analysis indicated that subjective norms had a large effect on preventive behavior ($f_{WH}^{2}$ = 0.821, $f_{OR}^{2}$ = 0.409), while the f^2^ of negative emotions had a medium effect on excessive preventive intention ($f_{WH}^{2}$ = 0.296, $f_{OR}^{2}$ = 0.192). And other variables supported by the test hypotheses had medium or small effects. Standardize root mean square residual (SRMR) was employed to evaluate model fit. The estimated SRMR value in the Wuhan group was 0.064; and the estimated SRMR value in other regions group was 0.056. Both values were lower than the cutoff value 0.08 [97]; hence our model satisfied a good fit.

### 4.6. Multi-Group Analysis

Several ways in PLS-SEM can be used in the multi-group analysis. Two common types for comparison between the two groups are the parametric significance test and the non-parametric significance test, and the latter is more applicable. SmartPLS 3.3.2 supports two methods of non-parametric significance tests, which are permutation and PLS-MGA. And permutation is strongly recommended by Hair et al. for comparison differences of parameters across two groups [86].

The results obtained from the multi-group analysis indicated there were no prominent differences between Wuhan and Other areas. Table 8 presented the detailed information of the multi-group analysis. This also meant that hypotheses H6a, H6b and H6c were not supported. Compared with other regions group, the influence of social media on the three mediators was not significantly stronger in the Wuhan group. Besides, according to the results of the Equal Mean Assessment step in MICOM, the values of excessive preventive intention, negative emotions, risk perception and subjective norms in the Wuhan group were significantly higher than those in other regions group, that is, Wuhan people had stronger excessive preventive intention than those in other regions group, and their perception of the other three factors was also stronger. However, there was no significant difference in mass media exposure and social networking services involvement, which indicated that there was no significant difference in media contact intensity between people in Wuhan and other regions.

### 4.7. Importance-Performance Map Analysis

**Figure 4:**
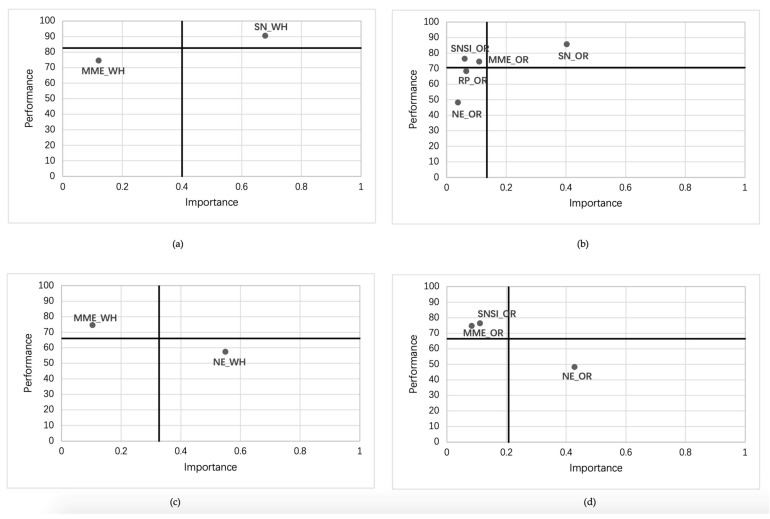
Results of IPMA. (**a**) IPMA for PB (Wuhan); (**b**) IPMA for PB (Other regions); (**c**) IPMA for EPI (Wuhan); (**d**) IPMA for EPI (Other regions).

The importance-performance map can be divided into four quadrants to further compare the performance of each dimension [98]. The creation of the four boundaries depends on the locating of the cross-hairs. In this study, the placement of the cross-hairs would be the overall means of the importance and performance ratings across all the factors in the present study [98]. For the Wuhan group and other regions group, the IPMA test was conducted with preventive behavior and excessive preventive intention as target constructs. After excluding the variables of total effects insignificant, the values of importance and performance were shown in Table 9. And the results of IPMA are were presented in Figure 4.

## 5. Discussion

The current study revealed that for other regions than Wuhan in the Chinese mainland, the assumption that mass media exposure would directly affect risk perception and negative emotions was not found, whereas the positive impact of mass media exposure on subjective norms was confirmed. The possible reason may be that after entering the stage of ongoing prevention and control, the media mainly adopts the reassurance frame for news reporting [52]. Moreover, the public is relatively more concerned about actions undertaken by the government and medical information [99]. Thereby the influence of mass media exposure on risk perception and negative emotions was weakened. The direct impact of social networking services involvement on risk perception had not been verified; however, the findings suggested that social networking services involvement indirectly affected risk perception through negative emotions, which was consistent with the previous study on Ebola [21]. Specifically, social networking services involvement would probably cause strong self-related emotions, and then promote increasing risk perception and preventive behavior at the personal level.

As expected, the results demonstrated that subjective norms and risk perception had significant effects on preventive behavior. However, there was no significant effect of negative emotions on preventive behavior. The findings implied that negative emotions had little effect on preventive behavior in the context of this study, which may be due to the specific stage of pandemic development. In the early stage of the pandemic, the virus had spread fast and wide, leading to a large number of unpredictable infections and deaths [100]. Besides, the public had little awareness of infectious diseases and the lacked pandemic prevention supplies, which would easily provoke negative emotions, such as public fear and anxiety [101]. Consequently, the public might pay more attention to preventive behavior and its related effectiveness information [102]. However, during the period of ongoing prevention and control, the pandemic had been controlled; moreover, after more than two months of the popularization of pandemic prevention knowledge, the public had been equipped with more relevant knowledge of the pandemic. Therefore, the impact of negative emotions on preventive behavior during this period was greatly weakened compared with the early stage of the pandemic. This may imply that the public’s protective behavior is more driven by rational factors than irrational factors under the ongoing pandemic prevention. The promotion of negative emotions including fear and anxiety is difficult to prompt the implementation of public protective behavior.

Meanwhile, IPMA results suggested that the impact of mass media exposure and social networking services involvement on preventive behavior was situated in the high performance and low importance quadrant, which also confirmed the view that the impact of media on individual protective behavior was limited [32]. In contrast, subjective norms were located in the quadrant of high performance and high importance, which indicated that preventive behavior was mainly affected by subjective norms. A possible explanation for this result may lie in the following three reasons. Firstly, as the pandemic situation has entered the stage of ongoing prevention and control, the public had learned enough knowledge of pandemic prevention through the media and had been implemented preventive measures for a period, and thus gradually formed a consensus of social norms. Therefore, the impact of subjective norms on preventive behavior has been greatly strengthened, and it takes precedence over risk perception and negative emotions. Secondly, the public’s previous experience of SARS prevention may reinforce the role of subjective norms. Personal experience reminds people of the risk more often and clearly [103]. Moreover, the previous similar disaster-related experience can enhance the ability of preventive behavior and risk perception [104]. Research has illustrated that the impact of personal norms on behavior is strongly reinforced by direct experience [105]. Many people in the Chinese mainland had experienced SARS, and because of the strong correlation between COVID-19 and SARS, it may arouse the public’s various experiences of pandemic preventive measures in the SARS era, which promotes the formation of social norms for pandemic prevention. However, what cannot be ignored is that the impact of previous disaster-related experience on risk perception is closely associated with the frequency and nature of disasters [106]. Furthermore, there are still some differences in the personal pandemic preventive measures between the SARS era and the period of the outbreak of COVID-19, such as the requirements of wearing face masks. Whether these specific experiential memories can be transferred directly and play a role is still controversial. Last and the most essential point is the influence of national culture. The predictive power of subjective norms on behavior varies in different national cultural contexts. A comparative study on the H1N1 self-protection behaviors of South Korea (collectivism) and the United States (individualism) and found that the predictive power of subjective norms on Korean samples was stronger than that of American samples [82]. In a typical collectivist culture, collectivists in the Chinese mainland are context-centered and tend to change their behavior according to the social environment [107]. Undoubtedly, the relationship between social norms and behaviors is stronger than that in individualistic culture [108]. However, for the Wuhan group, referring to the results of IPMA, it can be seen that the role of subjective norms is more prominent. The possible reason to explain the finding may be due to the earlier and much severer outbreak of the pandemic in Wuhan, and the generally acknowledged protective behavior norms were established earlier and implemented more thoroughly.

In terms of excessive preventive intention, the results of the Wuhan group were similar to those of other regions group. Excessive preventive intention was mainly affected by negative emotions, but risk perception did not play a significant role. This shows that excessive preventive intention is mainly caused by irrational factors if the protective behavior recommended by the government is effective and appropriate, and the result is in line with practical experience. According to the results of IPMA, negative emotions located in the quadrant of low performance high importance quadrant, which implied that under the theoretical framework and research background of this study, the influence of negative emotions on the willingness to excessive preventive intention was still limited, and the explanatory power of the existing models for excessive preventive intention was rather low. It is not ruled out that there are other factors not included in the influence of excessive preventive intention.

In addition, according to the results of MGA, no significant difference was identified in all path coefficients between the Wuhan group and other regions group, that is, there was no significant difference between the Wuhan area and other regions under the interpretation framework of this study. This may be because the difference in the severity of the pandemic was mainly reflected in the early and development stages of the pandemic. As the pandemic situation was under control, the impact of this difference gradually disappears.

## 6. Conclusions

In the context of ongoing prevention and control in the Chinese mainland, this study had established a model to explain the mechanism of media’s influence on preventive behavior and excessive preventive intention. Having used PLS-SEM to analyze the data, the results showed that mass media exposure had a significant positive impact on social networking services involvement and subjective norms, while preventive behavior was largely affected by subjective norms; social networking services involvement had a significant positive effect on negative emotions, and excessive preventive intention was mainly influenced by negative emotions. Overall, the importance of media to the two dependent variables was not as great as expected.

According to the severity of the pandemic, the respondents were divided into the Wuhan group and other regions group. Through multi-group analysis, there was no significant difference between the two groups. The above findings indicated that after the Chinese mainland entered the stage of ongoing pandemic prevention and control, mass media exposure can enhance the public’s preventive behavior through subjective norms, and subjective norms play a crucial role in this process. However, excessive preventive intention, to a large extent, is irrational behavioral decision-making, which is heavily influenced by negative emotions. Meanwhile, social networking services involvement played a role through the influence on negative emotions. Furthermore, during the pandemic, the impact of social media on public psychology did not differ in the severity of previous outbreaks.

What cannot be neglected is that there are still some limitations in this study. First of all, under the influence of the pandemic, offline questionnaire survey cannot be carried out as usual, data were collected only through the online questionnaire system, which to a certain extent results in the insufficient number of respondents who do not use the internet or use it less often; also, the number of elderly respondents is small, which has a certain impact on the wholeness of the respondent group. Secondly, based on the situation of normal pandemic prevention and control, cross-sectional data sets were used in this study, which could not reflect the dynamic changes of various factors in different stages of pandemic development.

Future research can be deepened in two directions. Firstly, this study found that subjective norms played an important role in preventive behavior, which differs from previous studies. The possible explanation for this result may be due to cultural differences. Therefore, follow-up research can conduct a cross-cultural comparison to investigate the differences caused by different social and cultural backgrounds. Secondly, in this study, the explanatory power of the model for excessive preventive intention was still weak, which implies that maybe there are other important variables and influencing paths which are not mentioned in this study. Thus, future research can further explore other important variables and influencing paths based on different stages of the pandemic.

## Figures and Tables

**Table 1:** Abbreviations, Definitions and Hypotheses of Variables

Variables	Abbreviations	Definitions	Corresponding Hypotheses
mass media exposure	MME	the amount of exposure that the public obtains information about the pandemic from the mass media, including television, newspaper, radio, news apps or websites and so on [26]	H1a H1b H1c H1d
social networking services involvement	SNSI	the public use of social media to interact and exchange information related to the pandemic with other social members [26]	H2a H2b H2c
risk perception	RP	the judgments people make when they are asked to characterize and evaluate hazardous activities and technologies [5]	H3a H3b
negative emotions	NE	The prompted negative affective associations with particular stimuli (COVID-19 pandemic) as well as deep cognitive reflection, such as fear and worry [27]	H4a H4b H4c
subjective norms	SN	a kind of pressure received from important others to or not to perform a behavior [28]	H5
preventive behavior	PB	a protective action undertook to reduce potential negative effects when people perceive that the risky situation is personally relevant [29].	-
excessive preventive intention	EPI	the public’s intention to implement preventive behaviors higher than the standards of official recommendations.	-

**Table 2:** Descriptive statistics of the variables in the model.

		Wuhan (N = 208)			Other Regions (N = 445)		
Constructs	Items	Mean	Standard Deviation	Average	Mean	Standard Deviation	Average
**Excessive preventive intention**	EPI1	4.380	1.721	4.337	4.170	1.768	3.867
	EPI2	4.350	1.741		3.760	1.721	
	EPI3	4.280	1.645		3.670	1.709	
**Mass media exposure**	MME1	4.270	2.342	4.066	4.100	2.157	3.904
	MME2	1.780	1.679		1.790	1.564	
	MME3	5.800	1.846		5.300	1.953	
	MME4	5.970	1.668		6.020	1.479	
	MME5	2.510	2.171		2.310	1.795	
**Negative emotions**	NE1	4.280	1.758	4.437	3.740	1.706	3.882
	NE2	4.780	1.704		4.180	1.751	
	NE3	4.530	1.716		3.880	1.703	
	NE4	4.140	1.760		3.540	1.684	
	NE5	4.100	1.660		3.530	1.689	
	NE6	4.790	1.651		4.420	1.853	
**Preventive behavior**	PB1	6.690	0.646	6.630	6.570	0.818	6.543
	PB2	6.620	0.898		6.600	0.720	
	PB3	6.710	0.647		6.580	0.772	
	PB4	6.530	0.952		6.450	0.903	
	PB5	6.570	0.739		6.490	0.835	
	PB6	6.660	0.776		6.570	0.770	
**Risk perception**	RP1	3.490	1.627	4.840	2.900	1.590	4.533
	RP2	4.710	1.806		4.490	1.815	
	RP3	5.670	1.358		5.570	1.402	
	RP4	5.490	1.458		5.170	1.470	
**Social networking services involvement**	SNSI1	5.270	1.853	4.798	5.270	1.646	4.683
	SNSI2	5.950	1.620		5.920	1.369	
	SNSI3	3.680	2.277		3.580	2.047	
	SNSI4	4.290	2.121		3.960	2.074	
**Subjective norms**	SN1	6.490	0.828	6.423	6.150	1.022	6.137
	SN2	6.320	0.921		6.080	1.096	
	SN3	6.460	0.779		6.180	1.007	

**Table 3:** Results of HTMT.

Relationships	Confidence Interval (95%) Bias Corrected	
	Wuhan	Other Regions
NE -> EPI	[0.428, 0.690]	[0.335, 0.523]
RP -> EPI	[0.102, 0.366]	[0.041, 0.197]
RP -> NE	[0.404, 0.650]	[0.344, 0.545]
SN -> EPI	[0.018, 0.096]	[0.013, 0.056]
SN -> NE	[0.108, 0.322]	[0.035, 0.131]
SN -> RP	[0.062, 0.313]	[0.084, 0.280]

**Table 4:** Results for reflective measurement models.

		Loadings		CR		Cronbach’s α		AVE		HTMT (HTMT Confidence Interval Does Not Include 1)	
Constructs	Type of Construct	Wuhan	Other Regions	Wuhan	Other Regions	Wuhan	Other Regions	Wuhan	Other Regions	Wuhan	Other Regions
Excessive preventive intention	Reflective			0.916	0.896	0.863	0.832	0.785	0.745	YES	YES
EPI1		0.846	0.697								
EPI2		0.898	0.933								
EPI3		0.912	0.937								
Negative emotions	Reflective			0.925	0.933	0.902	0.914	0.674	0.700	YES	YES
NE1		0.823	0.829								
NE2		0.884	0.894								
NE3		0.852	0.892								
NE4		0.888	0.835								
NE5		0.743	0.781								
NE6		0.720	0.782								
Risk perception	Reflective			0.898	0.874	0.833	0.784	0.746	0.699	YES	YES
RP2		0.855	0.823								
RP3		0.818	0.799								
RP4		0.915	0.884								
Subjective norms	Reflective			0.925	0.932	0.878	0.890	0.804	0.820	YES	YES
SN1		0.907	0.893								
SN2		0.851	0.894								
SN3		0.930	0.929								

**Table 5:** Results for formative measurement models.

		Weights		Loadings		VIFs		Convergent Validity	
Constructs	Type of Construct	Wuhan	Other Regions	Wuhan	Other Regions	Wuhan	Other Regions	Wuhan	Other Regions
**Mass media exposure**	Formative							0.754	0.785
MME1		0.478 **	0.379 ***	0.626 ***	0.491 ***	1.040	1.138		
MME3		0.403 *	0.355 **	0.653 ***	0.599 ***	1.113	1.165		
MME4		0.579 ***	0.752 ***	0.756 ***	0.800 ***	1.097	1.029		
**Preventive behavior**	Formative							0.806	0.773
PB1		−0.100 ^ns^	0.113 ^ns^	0.828 ***	0.780 ***	4.811	2.533		
PB2		0.392 ^ns^	0.176 ^ns^	0.911 ***	0.854 ***	3.180	3.407		
PB3		0.168 ^ns^	0.355 *	0.874 ***	0.914 ***	4.911	3.481		
PB4		0.239 ^ns^	0.380 **	0.847 ***	0.892 ***	2.526	3.336		
PB5		0.016 ^ns^	0.079 ^ns^	0.715 ***	0.864 ***	2.162	4.147		
PB6		0.403 ^ns^	0.036 ^ns^	0.905 ***	0.828 ***	3.247	3.218		
**Social networking services involvement**	Formative							0.901	0.837
SNSI1		0.449 ^ns^	0.443 ***	0.854 ***	0.840 ***	2.027	1.693		
SNSI2		0.517 *	0.638 ***	0.892 ***	0.916 ***	1.736	1.494		
SNSI3		−0.259 ^ns^	0.018^ns^	0.398 **	0.454 ***	2.050	2.375		
SNSI4		0.369 ^ns^	0.069^ns^	0.698 ***	0.502 ***	2.381	2.401		

Notes: * *p* < 0.05, ** *p* < 0.01, *** *p* < 0.001, ns = not significant (*p* > 0.05).

**Table 6:** Results of invariance measurement testing.

	Configural Invariance	Compositional Invariance		Partial Measurement Invariance	Equal Mean Assessment			Equal Variance Assessment			Full Measurement Invariance
Constructs		C = 1	Confidence Interval		Difference	Confidence Interval	Equal	Difference	Confidence Interval	Equal	
EPI	Yes	0.996	[0.995, 1.000]	Yes	0.337	[−0.166, 0.157]	No	−0.012	[−0.205, 0.178]	Yes	No
MME	Yes	0.981	[0.890, 1.000]	Yes	0.118	[−0.180, 0.182]	Yes	0.225	[−0.302, 0.286]	Yes	Yes
NE	Yes	1.000	[0.999, 1.000]	Yes	0.384	[−0.175, 0.162]	No	−0.064	[−0.207, 0.185]	Yes	No
PB	Yes	0.957	[0.869, 1.000]	Yes	0.130	[−0.165, 0.156]	Yes	−0.034	[−0.481, 0.453]	Yes	Yes
RP	Yes	0.998	[0.993, 1.000]	Yes	0.171	[−0.149, 0.151]	No	0.036	[−0.260, 0.245]	Yes	No
SNSI	Yes	0.975	[0.861, 1.000]	Yes	0.046	[−0.160, 0.154]	Yes	0.352	[−0.299, 0.271]	No	No
SN	Yes	0.999	[0.999, 1.000]	Yes	0.325	[−0.163, 0.154]	No	−0.453	[−0.409, 0.400]	No	No

**Table 7:** Results for structural models.

		Path Coefficient		T Statistics		Supported		R^2^		f^2^	
Hypothesis	Relationships	Wuhan	Other Regions	Wuhan	Other Regions	Wuhan	Other Regions	Wuhan	Other Regions	Wuhan	Other Regions
H3b	RP -> EPI	−0.048	−0.084	0.634 ^ns^	1.668 ^ns^	No	No	0.264	0.167	0.002	0.007
H4c	NE -> EPI	0.536	0.434	7.966 ***	9.641 ***	Yes	Yes			0.296	0.192
H1c	MME -> NE	0.090	0.075	1.019 ^ns^	1.538 ^ns^	No	No	0.067	0.083	0.007	0.005
H2b	SNSI -> NE	0.208	0.250	2.279 *	4.616 ***	Yes	Yes			0.038	0.057
H3a	RP -> PB	0.065	0.120	0.901 ^ns^	2.850 **	No	Yes	0.458	0.324	0.006	0.018
H4b	NE -> PB	−0.074	0.035	1.074 ^ns^	0.832 ^ns^	No	No			0.007	0.002
H5	SN -> PB	0.678	0.532	8.239 ***	9.744 ***	Yes	Yes			0.821	0.409
H1b	MME -> RP	−0.032	0.068	0.472 ^ns^	1.299 ^ns^	No	No	0.256	0.167	0.001	0.005
H2a	SNSI -> RP	0.134	0.093	1.548 ^ns^	1.803 ^ns^	No	No			0.019	0.008
H4a	NE -> RP	0.464	0.349	7.329 ***	6.896 ***	Yes	Yes			0.270	0.134
H1a	MME -> SNSI	0.418	0.407	4.948 ***	7.340 ***	Yes	Yes	0.175	0.166	0.212	0.199
H1d	MME -> SN	0.290	0.222	3.452 ***	4.324 ***	Yes	Yes	0.105	0.098	0.078	0.046
H2c	SNSI -> SN	0.066	0.149	0.782 ^ns^	2.484 *	No	Yes			0.004	0.020

Notes: * *p* < 0.05, ** *p* < 0.01, *** *p* < 0.001, ns = not significant (*p* > 0.05).

**Table 8:** Results of MGA.

Hypothesis	Relationships	Path Coefficient Difference (Wuhan-Other Regions)	PLS-MGA *p* Values	Permutation *p* Values	Supported
H6a	SNSI -> RP	0.041	0.719	0.672	No
H6b	SNSI -> NE	−0.042	0.687	0.668	No
H6c	SNSI -> SN	−0.083	0.420	0.391	No
	MME -> NE	0.014	0.879	0.882	-
	MME -> RP	−0.101	0.247	0.253	-
	MME -> SNSI	0.011	0.884	0.922	-
	MME -> SN	0.068	0.477	0.451	-
	NE -> EPI	0.102	0.206	0.213	-
	NE -> PB	−0.109	0.179	0.141	-
	NE -> RP	0.115	0.161	0.183	-
	RP -> EPI	0.036	0.688	0.706	-
	RP -> PB	−0.056	0.489	0.489	-
	SN -> PB	0.146	0.147	0.163	-

Notes: Significance level is 0.05.

**Table 9:** Values of Importance and Performance.

			Importance	Performance
PB	Wuhan	MME	0.121	74.527
		SN	0.679	90.612
		Average	0.400	82.570
	Other regions	MME	0.109	74.643
		NE	0.038	48.225
		RP	0.066	68.421
		SNSI	0.061	76.392
		SN	0.403	85.675
		Average	0.135	70.671
EPI	Wuhan	MME	0.104	74.527
		NE	0.549	57.419
		Average	0.327	65.973
	Other regions	MME	0.083	74.643
		NE	0.428	48.225
		SNSI	0.111	76.392
		Average	0.207	66.420

## Appendix A

Main items of the questionnaire

**Part I**

**Mass media exposure**

How often a respondent reads/watches the news about COVID-19 from different mass media channels:

- MME1—television news programs
- MME2—newspapers
- MME3—news apps on mobile phones/news websites
- MME4—media’s public platforms on microblogs/WeChat
- MME5—radio
- Global single item--Generally speaking, how often do you get the information of COVID-19 through the mass media (including the above-mentioned various forms of mass media) during the pandemic?

**Social networking services involvement involvement**

How often concerning activities about COVID-19 issue on social network sites such as Weibo and WeChat:

- SNSI1—talk about
- SNSI2—pay attention to
- SNSI3—post messages
- SNSI4—relay messages
- Global single item—Generally speaking, how often do you get the information of COVID-19 through social media platforms (such as WeChat, micro-blog, Zhihu, Douban, etc.) during the pandemic?

**Risk perception**

Please indicate your level of agreement to the following statements.

- RP1—I am very likely to get the COVID-19.
- RP2—If I get the COVID-19, it will be severe.
- RP3—If I get the COVID-19, it will be risky.
- RP4—If I had the COVID-19, I would not be able to manage daily activities.

**Negative emotions**

Please indicate your level of agreement to the following statements.

- NE1—I am afraid of COVID-19.
- NE2—I am frightened by COVID-19.
- NE3—I scared of COVID-19.
- NE4—I am always afraid that I am infected with COVID-19.
- NE5—I usually feel at high risk for developing COVID-19.
- NE6—If I have a body discomfort, I must know what is the cause during the outbreak.

**Subjective norm**

Please indicate your level of agreement to the following statements.

- SN1—My family members have taken preventive measures against the COVID-19.
- SN2—My close friends have taken preventive measures against the COVID-19.
- SN3—The general public around me has taken preventive measures against the COVID-19.

**Preventive Behavior**

How likely are you in undertaking the following preventive measures?

- PB1—Minimize social activities; avoid infected areas; avoid crowded public places.
- PB2—Wear a single-use medical face mask when visiting public places, or taking public transport; wear a surgical mask when visiting a fever clinic.
- PB3—Keep your hands clean and wash your hands frequently; minimize contacts with objects in public places.
- PB4—Refrain from touching your mouth, nose, and eyes with unwashed hands; cover your mouth and nose with your elbow when sneezing or coughing.
- PB5—Monitor your health conditions; wear a face mask and visit a nearby clinic for medical help when any suspicious symptom comes up.
- PB6—Ensure your home is adequately ventilated.
- Global single item—During the pandemic, I took personal preventive measures according to the above preventive measures.

**Excessive preventive intention**

Please indicate your level of agreement with the following statements.

- EPI1—I think the above preventive measures are not enough to prevent COVID-19.
- EPI2—I tend to take more stringent preventive measures besides the above preventive measures.
- EPI3—I don’t think the above preventive measures can guarantee my protection against COVID-19.

**Part II**

- Your gender
- Your age
- Your education level
- Your current occupation
- During the pandemic, which city do you live in?
- Do you belong to the following groups during the pandemic? (Confirmed cases/Suspected cases/Close contacts/None of the above)
- During the pandemic, do you work in the front line of fighting the pandemic?
